# SEOM clinical guidelines for the diagnosis and treatment of gastroenteropancreatic and bronchial neuroendocrine neoplasms (NENs) (2018)

**DOI:** 10.1007/s12094-018-1980-7

**Published:** 2018-12-07

**Authors:** E. González-Flores, R. Serrano, I. Sevilla, A. Viúdez, J. Barriuso, M. Benavent, J. Capdevila, P. Jimenez-Fonseca, C. López, R. Garcia-Carbonero

**Affiliations:** 1 Department of Medical Oncology, Hospital Universitario Virgen de las Nieves, Granada, Spain; 2 Department of Medical Oncology, Hospital Reina Sofía, Córdoba, IMIBIC, CIBERONC, Córdoba, Spain; 3 Department of Medical Oncology, Instituto de Investigaciones Biomédicas de Málaga (IBIMA)/Hospitales Universitarios Regional y Virgen de la Victoria de Málaga, Málaga, Spain; 4 Department of Medical Oncology, Complejo Hospitalario de Navarra (CHN), OncobionaTras Unit, Navarrabiomed, IdiSNA, Pamplona, Spain; 5 Division of Cancer Sciences, School of Medical Sciences, Faculty of Biology, Medicine and Health, University of Manchester, Manchester, UK; 6 Department of Medical Oncology, The Christie NHS Foundation Trust, Manchester, UK; 7 Department of Medical Oncology, Hospital Universitario Virgen del Rocío, Instituto de Biomedicina, Seville, Spain; 8 Department of Medical Oncology, Vall Hebron University Hospital, Vall Hebron Institute of Oncology (VIHO), Barcelona, Spain; 9 Department of Medical Oncology, Hospital Universitario Central de Asturias, Oviedo, Spain; 10 Department of Medical Oncology, Hospital Universitario Marqués de Valdecilla, Santander, Spain; 11 Department of Medical Oncology, Hospital Universitario 12 de Octubre, IIS imas12, UCM, CNIO, CIBERONC, Av. de Córdoba, s/n, 28041 Madrid, Spain

**Keywords:** Guidelines, Neuroendocrine tumors, Neuroendocrine neoplasms, Gastroenteropancreatic, Lung, Diagnosis, Therapy

## Abstract

NENs are a heterogeneous family of tumors of challenging diagnosis and clinical management. Their incidence and prevalence continue to rise across all sites, stages and grades. Although improved diagnostic techniques have led to earlier detection and stage migration, the improved prognosis documented over time for advanced gastrointestinal and pancreatic neuroendocrine tumors also reflect improvements in therapy. The aim of this guideline is to update practical recommendations for the diagnosis and treatment of gastroenteropancreatic and lung NENs. Diagnostic procedures, histological classification and therapeutic options are briefly discussed, including surgery, liver-directed therapy, peptide receptor radionuclide therapy, and systemic hormonal, cytotoxic or targeted therapy, and treatment algorithms are provided.

## Introduction

The incidence of NENs has increased 6.4-fold over the past four decades, from 1.09 (1973) to 6.98 (2012) new cases per 100,000 inhabitants annually [1]. Per primary tumor site, the incidence rates were 3.56/10^5^ in gastroenteropancreatic (GEP), 1.49/10^5^ in the lung and 0.84/10^5^ in neuroendocrine tumors (NETs) of unknown primary site. Survival for all NENs has improved, particularly for distant-stage pancreatic and gastrointestinal NETs (panNETs and GINETs), although it widely varies by primary tumor site, proliferative index (ki67 or mitotic index), histological differentiation and stage [1, 2]. According to the SEER data, rectum (24.6 years) and appendix (> 30.0 years) NETs had the best prognosis, while panNETs (3.6 years) and lung NETs (5.5 years) had the worst survival. Survival by grade also widely ranged from 16.2 and 8.3 years for grade G1 and G2 to 10 months for high-grade tumors. About 20–25% of NENs are functioning tumors, that is, they are associated with a clinical syndrome due to excessive hormone production (carcinoid syndrome, Zollinger–Ellison syndrome, etc.). This proportion is decreasing with time due to earlier diagnosis and improved symptomatic and antineoplastic therapy [3]. They are generally diagnosed in the fifth decade of life, and about 5% of them are associated with hereditary predisposition syndromes. NENs are therefore a complex, heterogenous family of tumors of challenging clinical management. In this manuscript, we aim to provide synthetical and practical guidelines regarding diagnostic procedures and therapeutic options for the multidisciplinary management of NENs of GEP or lung origin. Available medical literature was reviewed according to main topics of disease management and classified by scientific levels of evidence and grades of clinical recommendation (Table 1).

**Table 1:** Levels of evidence and grades of recommendation

Levels of evidence	Grades of recommendation
I. Evidence from at least one large randomized, controlled trial of good methodological quality (low potential for bias) or meta-analyses of well-conducted randomized trials without heterogeneity	A. Strong evidence for efficacy with a substantial clinical benefit, strongly recommended
II. Small randomized trials or large randomized trials with a suspicion of bias (lower methodological quality) or meta-analyses of such trials or of trials with demonstrated heterogeneity	B. Strong or moderate evidence for efficacy but with a limited clinical benefit, generally recommended
III. Prospective cohort studies	C. Insufficient evidence for efficacy or benefit does not outweigh the risk or the disadvantages (adverse events, costs,…), optional
IV. Retrospective cohort studies or case–control studies	D. Moderate evidence against efficacy or for adverse outcome, generally not recommended
V. Studies without control group, case reports, experts opinions	E. Strong evidence against efficacy or for adverse outcome, never recommended

## Diagnostic procedures

The diagnosis of NENs may require clinical, biochemical, pathological, radiological, nuclear medicine or endoscopic procedures, depending upon primary tumor site, tumor stage and clinical presentation including hormonal syndromes [3]. Besides a comprehensive medical history, physical examination and laboratory tests (including hematological, liver and renal function parameters), the following procedures are recommended for an adequate diagnosis of NENs:

- Chromogranin A (well-differentiated NETs) (III,B) or neuron-specific enolase (NSE) [poorly differentiated neuroendocrine carcinomas (NECs)] (I, C).
- Urinary 5-hydroxyindoleacetic acid (5-HIAA) (carcinoid syndrome); gastrin ± secretin test (gastrinomas); insulin/glucose ratio, proinsulin, C peptide (insulinomas), glucagon, VIP and others depending upon clinical symptoms (IV, C).
- Histopathological report should include the WHO classification and TNM staging, as well as immunohistochemistry staining including ki67 and general neuroendocrine markers (chromogranin A, synaptophysin and NSE). Specific markers are not mandatory and should only be performed if clinically indicated (insulin, glucagon, etc.) (I, A).
- Somatostatin receptor imaging: ^68^Gallium-DOTA-TOC/-NOC/-TATE positron emission tomography (PET) (preferred if available) or somatostatin receptor scintigraphy (octreoscan) [4] (III, B).
- Dynamic CT scan or MRI of the abdomen.
- Chest X-ray. A thoracic CT scan may be considered in poorly differentiated tumors, colon primaries or those in whom surgery of liver metastasis is being considered and is also mandatory in lung NENs.
- Genetic counseling in hereditary predisposition syndromes (MEN-1, von Hippel-Lindau disease, tuberous sclerosis, and neurofibromatosis, among others).

Depending on the clinical presentation and site of the primary tumor, other evaluations such as endoscopic procedures, 18-Fluorodeoxyglucose-(FDG-) PET, N-terminal pro-brain natriuretic peptide (NT-pro-BNP) and echocardiogram (carcinoid syndrome), brain CT or bone scan may be performed.

## Classification and staging systems

The 2010 WHO classification of digestive tumors is the most widely accepted classification of GINENs by the scientific community. It stratifies these tumors based on histological differentiation, ki67 and mitotic index into three major prognostic groups: G1 and G2 NETs and G3 NECs [5, 6]. However, in 2017, the WHO classification of Endocrine Tumors was updated, and this includes panNETs but not GINETs. This subdivides G3 NENs into well-differentiated tumors (G3 NETs) and poorly differentiated carcinomas (G3 NECs) [7].

In NET of the lung, the 2015 WHO classification [8] is the most widely used, and defines four categories (typical carcinoid, atypical carcinoid, large cell NEC and small cell NEC) based on morphological features, the mitotic count and the presence of necrosis. The cutoff point of ki67 is under discussion and not required for tumor classification (Table 2 summarizes the main features of the WHO classification by tumor site).

**Table 2:** WHO classifications of pancreatic, gastrointestinal and lung neuroendocrine neoplasms

Pancreatic NENs WHO 2017	Gastrointestinal Tract NENs WHO 2010	Lung NENs WHO 2015
Well differentiated		
PanNET G1 ki67 < 3%; < 2 mit/10HPF PanNET G2 ki67 3–20%; 2–20 mit/10HPF PanNET G3 ki67 > 20%; > 20 mit/10HPF	NET G1 ki67 ≤ 2%; < 2 mit/10HPF NET G2 ki67 2–20% and/or 2–20 mit/10HPF	Typical carcinoid < 2 mit/10HPF; no necrosis Atypical carcinoid 2–10 mit/10HPF and/or foci of necrosis

Poorly differentiated		
PanNEC G3 ki67 > 20% > 20 mit/10HPF Small cell type Large cell type	NEC G3 ki67 > 20% and/or > 20 mit/10HPF Small cell type Large cell type	NEC small cell type (also combined small cell NEC) > 10 mitoses/10HPF NEC large cell type (also combined large cell NEC) > 10 mitoses/10HPF Combined small cell NEC > 10 mitoses/10HPF

Mixed neuroendocrine		
MiNEN: mixed neuroendocrine-nonneuroendocrine neoplasm	MANEC: mixed adeno-neuroendocrine carcinoma	

The final grade is determined based on whichever index (Ki67 or mitotic) places the tumor in the highest grade category

*PanNENs* pancreatic neuroendocrine neoplasms, *PanNET* pancreatic neuroendocrine tumor, *NET* neuroendocrine tumor, *NEC* neuroendocrine carcinoma

Probably, the inclusion of genetic alterations in the different classifications may help in the future to further characterize and stratify the prognosis of NENs.

Local, regional and distant extent of disease should be classified according to the American Joint Committee on Cancer/Union for International Cancer Control (AJCC/UICC) TNM staging system (8th edition).

## Therapy

### Grade 1–2 GEP and bronchial NETs (Fig. 1a, b) (Table 3)

#### Surgery

Surgery is the only potentially curative therapeutic strategy in these patients. Radical oncological surgery, even in cases with metastatic disease if complete resection is deemed feasible, is indicated except for small (< 1 cm) carcinoids of the stomach, duodenum, appendix or rectum, and small pancreatic insulinomas, in which more conservative surgical or endoscopic resections may be appropriate given their low risk of metastasis [9]. No adjuvant therapy is recommended after complete resection of well-differentiated (G1/G2) NETs (IV, C).

**Fig. 1:**
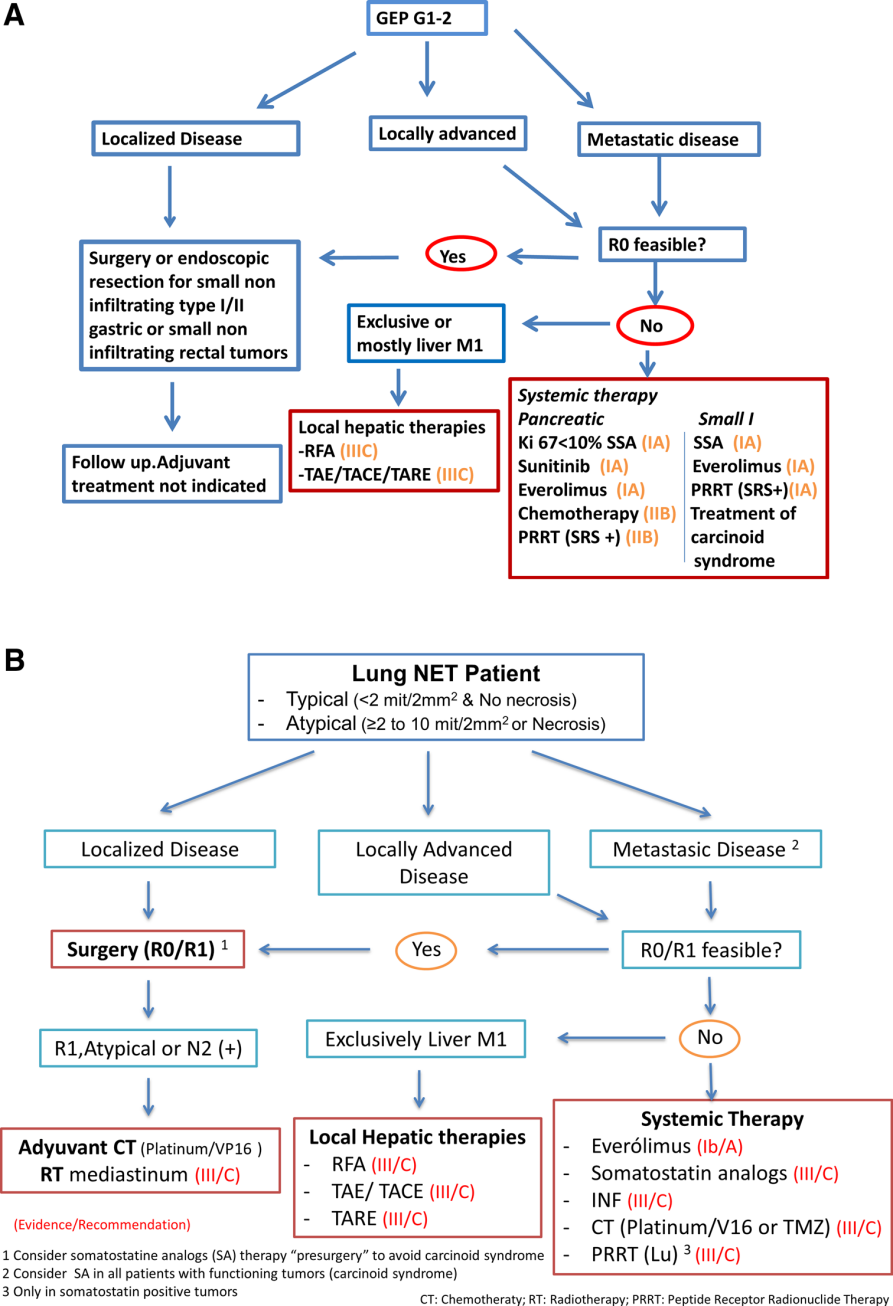
**a** Therapeutic algorythm for G1-2 GEP NETs. **b** Therapeutic algorythm for typical and atypical Lung NETs

**Table 3:** Summary of randomized phase III trials of systemic therapy in G1–2 NETs (J. Capdevila)

Study	*N*	Schedule	Primary endpoint	Secondary endpoints
Moertel CG [9]	105	STZ + DOXO vs. STZ + 5FU vs. Chlorozotocin	RR 69% vs. 45% vs. 30%	mPFS 20 months (STZ-Doxo) vs. 6.9 months (STZ-5FU) mOS 2.2 years (STZ-Doxo) vs. 1.4 years (STZ-5FU)
PROMID [15]	85	Octreotide LAR 30 mg vs. placebo	TTP 14.3 months vs. 6 months; HR 0.34; 95% CI 0.20–0.59, *p* = 0.000072)	
CLARINET [16]	204	Lanreotide Autogel 120 mg vs. placebo	mPFS 32.8 months vs. 18 months; HR 0.47; 95% CI 0.30–0.73, *p* = 0.001	Progression-free survival at 24 months: 65.1% vs. 33%
NETTER-1 [24]	229	177Lu-Dotatate vs. octreotide LAR 60 mg	mPFS 28.4 months vs. 8.5 months; HR 0.214; 95% CI 0.139–0.331, *p* < 0.0001	RR 18% vs. 3%
SU1111 [21]	171	Sunitinib 37.5 mg qd vs. placebo	mPFS 11.4 vs. 5.5 months; HR 0.42; 95% CI 0.26–0.66, *p* < 0.001	RR 9% mOS 38.8 vs. 29.1 months; HR 0.73; 95% CI 0.50–1.06; *p* = 0.094
RADIANT-3 [22]	410	Everolimus 10 mg qd vs. placebo	mPFS 11.0 vs. 4.6 months; HR 0.35; 95% CI 0.27–0.45, *p* < 0.001	RR 5% mOS 44 vs. 37.7 months; HR 0.94; 95% CI, 0.73–1.20; *p* = 0.30
RADIANT-4 [23]	302	Everolimus 10 mg qd vs. placebo	mPFS 11.0 vs. 3.9 months, respectively, HR 0.48; 95% CI 0.35–0.67, *p* < 0.00001	RR 2% mOS not reached (HR 0·64; 95% CI 0·40–1·05, one-sided *p* = 0.037, whereas the boundary for statistical significance was 0·0002)
RADIANT-2 [9]	429	Everolimus 10 mg qd + octreotide LAR 30 mg monthly vs. octreotide LAR 30 mg monthly	mPFS 16.4 vs. 11.3 months; HR 0.77, 95% CI 0.50–1.00, *p* = 0.026	RR 2% mOS 29.2 vs. 35.2 months (HR 1.17; 95% CI, 0.92–1.49, *p* = ns)

*N* number of patients, *STZ* streptozocin, *Doxo* doxorubicin, *5FU* 5-fluorouracil, *RR* response rate, *qd* once every day, *mPFS* median progression-free survival, *mOS* median overall survival, *HR* hazard ratio, *ns* non-significant

Major cytoreductive surgery with palliative purposes may be indicated when resection of > 90% tumor burden is feasible, especially for patients with hormonal syndrome refractory to medical therapy. Besides the significant clinical benefit in cases with functioning tumors, there are some retrospective data that suggest an impact in terms of survival [10] (IV, B).

In patients with unresectable metastatic disease, palliative surgery may be considered in cases with symptomatic primary tumor, mainly in patients with small bowel NETs. Perioperative prophylactic therapy with somatostatin analogues (SSA) is mandatory in functional tumors to prevent carcinoid crisis. Finally, liver transplantation is still controversial (44–52% 5-year overall survival (OS) rates) but may be considered in highly selected young patients (< 55 years) with well-differentiated liver-only metastases, resected primary tumor and low ki67 index (< 5%), who demonstrated a stable disease status for at least 6 months before organ transplantation [11] (IV, C).

#### Locoregional therapy

In patients who are not suitable candidates for surgery, regional control of liver metastases may be achieved (50–80% 5-year OS in small retrospective series) by different ablative techniques such as laparoscopic or percutaneous radiofrequency or laser ablation, and cryotherapy, among others [12, 13]. Other locoregional approaches include embolization of the hepatic artery by particles with or without cytotoxic agents (TACE/TAE) or radioactive microspheres (TARE). These therapies may be considered as an alternative approach to systemic therapies, especially for patients with symptomatic/functioning high liver tumor burden. Doxorubicin and mitomycin C are commonly used agents in this context, although adequately sized randomized studies that properly evaluate the benefit–risk ratio of chemoembolization with that of mechanical embolization are still lacking. Clinical responses have been reported in up to 80% of the patients, and radiological responses in about 50%. Embolization is contraindicated in patients with portal-vein thrombosis, liver insufficiency, biliary obstruction or prior Whipple procedure. Radioembolization is an alternative technique of liver-directed therapy (most commonly with yttrium-90 microspheres) with similar clinical results, but prospective randomized trials comparing it to the other liver-directed modalities are lacking [14] (III, C).

#### SSAs and interferon

SSAs are the first choice of therapy for symptom control in functional GEP–NETs (70–80% of patients experience resolution of diarrhea or flushing, and about 40% achieve biochemical response) (III, A) and they also have antiproliferative activity as demonstrated in two placebo-controlled randomized trials. (I, A) The PROMID study enrolled 85 patients with G1 metastatic midgut NETs, which were randomized to receive octreotide LAR (30 mg/28 days) or placebo. Time to tumor progression was significantly longer in the octreotide LAR group (14.3 months) as compared to patients treated with placebo (6 months) [HR 0.34; 95% confidence interval (CI) 0.20–0.59, *p *< 0.001]. The greatest effect was observed in patients with low hepatic tumor load and resected primary tumor [15]. No difference was observed, however, in OS among study arms. More recently, the CLARINET study [16] included 204 patients with non-functioning GEP–NETs and ki67 < 10% (45% pancreatic, 36% midgut, 7% hindgut and 13% of unknown primary), that were randomly allocated to receive lanreotide autogel (120 mg/28 days) or placebo [16]. Treatment with lanreotide significantly prolonged progression-free survival (PFS) over placebo (median not reached with lanreotide vs. 18 months with placebo, HR 0.47, *p *< 0.001). Of note, patients with high liver involvement (> 25%) also benefited from lanreotide. Currently recommended antiproliferative doses are octreotide LAR 30 mg or lanreotide autogel 120 mg every 28 days. Lower starting doses may be used for syndrome control, and then be titrated as needed. Short-acting subcutaneous octreotide may be necessary as rescue treatment for carcinoid syndrome exacerbations. Doses above 120 mg for lanreotide autogel or 30 mg for octreotide LAR are off-label but can sometimes be needed in selected cases. Adverse effects of SSA include malabsorption, hypo or hyperglycemia, hypothyroidism, pain and erythema at the site of injection, hypersensitivity reactions and cholelithiasis on long-term use.

Interferon has some activity in terms of symptomatic control of the hormonal syndrome, but adverse effects limit its widespread use (particularly fatigue, depression, liver toxicity, flu-like syndrome and myelosuppression). Although it may have some antiproliferative activity too, this has not been definitively proven [17], and it is therefore generally indicated after failure of other therapeutic options (III, C).

#### Chemotherapy

Systemic chemotherapy is indicated in progressive or bulky advanced panNETs and in G3 NENs. Chemotherapy may be considered in NETs of other sites (lung, thymus, stomach, colon, and rectum) in certain circumstances (e.g., ki67 in the upper G2 range, rapidly progressive disease and/or after failure of other therapies, particularly if somatostatin receptor imaging is negative). In patients with small intestinal primaries, chemotherapy plays a minor role and is generally reserved for patients with progressive disease along with other strategies (III, D).

Chemotherapy combinations most frequently used include streptozocin with either 5-FU or doxorubicin, with overall response rates (ORRs) of 45–69% in older trials (II,B) and 28–42% in more recent ones, and PFS ranging from 16 to 23 months [18]. Other active agents include dacarbazine or temozolomide, Recently, results of a randomized phase II trial showed an increase in progression-free survival (PFS) (from 14.4 to 22.7 months, HR 0.58, *p *= 0.023) and in survival (38 months vs. not reached, HR 0.41, *p *= 0.012) with similar response rates (27.8% vs. 33.3%) for the combination of temozolamide and capecitabine as compared to temozolamide alone in panNETs [19] (II, B).

No biomarkers may be currently recommended for routine clinical practice to predict response to chemotherapy. The use of MGMT deficiency as a predictive biomarker of temozolomide activity has not been demonstrated in prospective studies and is not considered standard.

No randomized studies have been performed in lung NETs (typical and atypical carcinoids). Chemotherapy is an option after failure of SSA and everolimus, or in patients with rapidly growing tumors. Temozolomide has been explored in non-randomized studies as well as in combination with oxaliplatin, with PFS ranging from 5 to 20 months [20] (III, B).

#### Targeted agents

Everolimus and sunitinib are approved worldwide for the treatment of advanced well-moderately differentiated NETs based on positive results of placebo-controlled phase III clinical trials. Sunitinib has demonstrated in an international, placebo-controlled phase III clinical study, significantly increased PFS in patients with advanced panNETs (median PFS 11.4 vs. 5.5 months; HR 0.42; 95% CI 0.26–0.66, *p* < 0.001) (I, A). Recent updated data showed a trend towards an improved OS in favor of sunitinib, with almost 10 months median survival increase compared with placebo (38.6 vs. 29.1 months, HR 0.73; 95% CI 0.50–1.06, *p *= 0.094) [21]. The RADIANT-3 trial randomized patients with advanced well-moderately differentiated panNETs to receive everolimus or placebo demonstrating an increase of PFS in favor of everolimus (11.0 vs. 4.6 months; HR 0.35; 95% CI 0.27–0.45, *p* < 0.001). A follow-up reported no significant improvement in OS, but this may be attributed to the fact that > 70% of patients in the placebo arm crossed over to everolimus upon disease progression [22]. The RADIANT-4 trial confirmed the everolimus effectiveness in non-functioning NETs of lung or gastrointestinal origin. PFS was significantly increased with everolimus as compared to placebo (11.0 vs. 3.9 months, respectively, HR 0.48; 95% CI 0.35–0.67, *p* < 0.00001) [23]. The results obtained by the RADIANT-3 and -4 trials led to the approval of everolimus by regulatory authorities for the treatment of advanced and progressive, well-moderately differentiated, functioning and non-functioning panNETs and for non-functioning gastrointestinal and lung NETs (I, A).

#### Metabolic therapy

Patients with advanced disease and a positive somatostatin-receptor imaging may be considered for peptide receptor radionuclide therapy (PRRT). PRRT primarily utilizes one of two radioisotopes, yttrium-90 (^90^Y) or lutetium 177(^177^Lu), linked to a SSA via the chelating agent 1,4,7,10-tetraazacyclo-dodecane-1,4,7,10-tetraacetic acid (DOTA).

The phase III NETTER-1 study, conducted in patients with midgut NETs progressive to standard-dose SSA, has shown that lutetium (^177^Lu)-DOTATATE, compared to high doses of octreotide, significantly increases the ORR (18 vs. 3%; *p *< 0.001) and PFS (28.4 vs. 8.5 months; HR 0.21, 95% CI 0.14–0.33, *p *< 0.0001), with a trend towards improved OS, although data are still immature for definitive conclusions in this regard (median not achieved vs. 27.4 months, HR 0.46, 95% CI 0.14–1.5). In addition, 177Lu-DOTATATE was able to improve the subjective measurement of patient’s quality of life in multiple relevant domains, such as global health status, physical functioning, role functioning, fatigue, diarrhea, pain, disease-related worries and body image. With limited median follow-up, 14 months, the most common grade 3–4 toxicities were lymphopenia (9%) and emesis (7%), with no renal toxicity detected [24].

Large series from reference European centres have reported significant PRRT activity in NETs of primary tumor sites other than the midgut. In this series, the ORR was 30%, PFS was 40 months, OS was 46 months. ORRs were higher in patients with gastrinomas, insulinomas, VIPomas and non-functioning panNETs than in carcinoid tumors. In multivariate analysis, uptake on the OctreoScan (*p *< 0.01), and Karnofsky performance status > 70% (*p *< 0.05) were prognostic factors for predicting tumor remission [25].

Therefore, PRRT is indicated in patients with well-differentiated, metastatic, unresectable midgut NETs, in progression to SSA and with positive somatostatin receptors (grade 2–4 scale Krenning). It is also required for the patient to have a good performance status (ECOG < 2), and an adequate renal, hepatic and bone marrow function. (I, A) Its administration to tumors of other primary sites may also be considered although the evidence to support it is not derived from randomized trials (II, B). The appropriate timing of this therapeutic intervention remains to be elucidated.

### Grade 3 NENs (Fig. 2)

Malignancies with a ki67 > 20% and poor differentiation are considered G3 NEC. Well-differentiated tumors with a proliferation index > 20% are called G3 NET by the NEN community [although this category only applies to panNETs in WHO 2017 (Table 2)] [26].

**Fig. 2:**
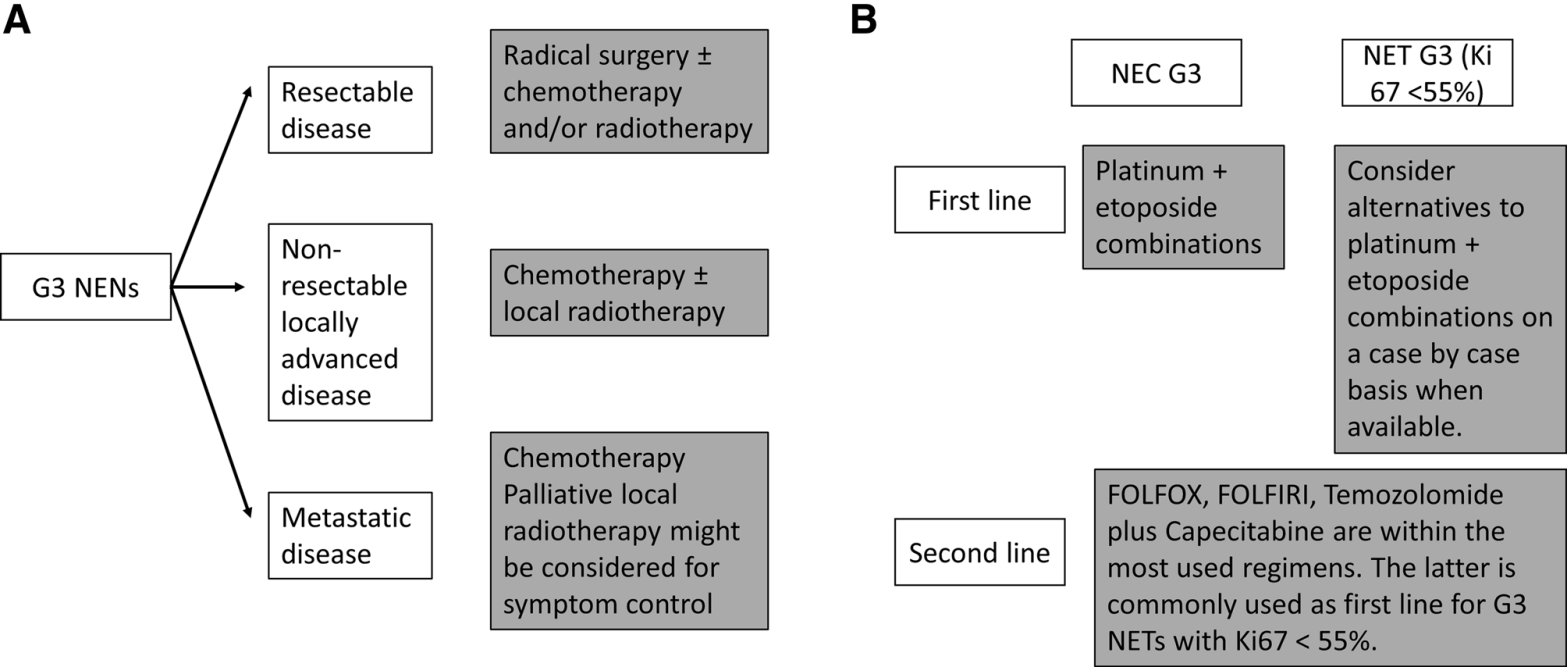
Therapeutic algorythm for G3 NENs

Localized disease is usually managed with surgery (IV, C). However, retrospective series indicate that this approach alone is rarely curative and suggest improved results with adjuvant chemotherapy. Radiotherapy may be used as consolidation therapy in this scenario too, and as an alternative to surgery in certain primary sites (i.e., esophageal NECs) or in the setting of non-resectable locally advanced disease. However, the management of NECs with non-resectable or metastatic advanced disease is still based on systemic cytotoxic chemotherapy (III, B). Current treatments are based on data from small cell lung cancer, most commonly treated with cisplatin/carboplatin–etoposide. First-line treatment for NECs has remained unchanged since the early 1990s, when high ORRs were reported with etoposide–platinum combination (41–67%). In addition, a number of small retrospective series have published results of other chemotherapy regimens (temozolomide-based, taxane-based, 5-FU-based, or topotecan/irinotecan-based) after failure of platinum–etoposide therapy in patients with NECs (IV, C) [27].

The marginal benefit observed in two retrospective series with first-line platinum combination in NENs with ki67 between 20 and 55% and in G3 NETs has led many doctors in specialized NET centres to recommend non-platinum combinations for this subgroup, such as temozolomide and capecitabine [28] (IV, C). This alternative, orally administered regimen has the added value of its improved tolerance.

## Follow-up

There is no consensus regarding optimal follow-up of resected NENs. A web-based practice survey targeted at NET health care providers in Australia, New Zealand, Canada and the US illustrated the great variation among professionals in follow-up practices and highlights the need for quantitative research to provide solid evidence to base follow-up guidelines to the pattern of recurrence in NENs [29].

Based on expert opinion, resected well-differentiated G1-2 NENs shall be followed-up every 6–12 months for the first 2–3 years after surgery, and then every 12–24 months up to 10 years. Follow-up intervals shall be shorter in patients with G3 NECs (every 3–6 months during the first 3 years, every 6–12 months thereafter). This follow-up must include a clinical evaluation (especially in functioning tumors), a physical examination, biochemical markers and imaging techniques (V, C). General tumor markers may be considered in all patients (chromogranin A in G1–2 NETs or NSE in G3 NECs), even though their value in follow-up is still to be proven, and specific markers shall be performed only in patients with hormone syndromes (5HIAA, insulin, gastrin…) (III, B). Regarding imaging techniques, an abdominal CT scan or MRI is recommended to be performed at least annually during the first 3 years, and biannually thereafter up to 10 years following surgery (V, C). Chest imaging or functional imaging is not recommended unless clinically indicated but is mandatory in lung NEN patients.

There are certain low-risk subgroups in which no follow-up is required, such as small (< 2 cm) G1 node-negative panNETs, node-negative insulinomas, incidental G1 T1–2 node-negative midgut NETs, small (< 1 cm) G1 appendiceal NETs, and G1 T1 node-negative rectal NETs (V, C).

In patients with advanced disease who are receiving active therapy, follow-up visits will depend on the tolerability, expected toxicity, concomitant diseases, symptom control (including hormonal syndrome), tumor kinetics and response to treatment (V, C). Visits may be done every 6 months for G1, slow growing tumors, in clinically stable patients under low-toxic therapies (i.e., SSA). These intervals shall be shortened, however, for patients with faster tumor growth rates or receiving more toxic agents. Radiological evaluation of the disease should be done every 3–6 months, most commonly by CT scan. The global heart function must be controlled in cases of possible carcinoid cardiac disease. However, clinical judgement must be applied and each patient individually assessed.
